# Diseases of the Stomach

**Published:** 1898-06

**Authors:** 


DISEASES OF THE STOMACH. Their Special Pathology, Diagnosis and Treatment, with Sections on Anatomy, Physiology, Analysis of Stomach Contents, Dietics, Surgery of the Stomach, etc. In Three Parts. By Jno. C. Hemmeter. M. B., M. D., Ph. D., Clinical Professor of Medicine at the Baltimore Medical College; Consultant to the Maryland General Hospital, etc. With many Original Illustrations, many of which are in Colors. 788 pages. Price, cloth, $6. Published by P. Blackiston, Son & Co., Philadelphia. 1897.

This is one of those excellent works, which, being devoted to a single subject and written by an able author, leaves little to be desired.

The subject is the most important that presents itself to the general practioner, and is an indisputable field of study to every specialist. It has been said that "the blood is the life," but we must invariably, in practice, go further, and fully realize that good blood is only to be produced by good digestion and assimilation of food, and that bad digestion or assimilation is a bad disease, a morbid entity, in most cases.

The scientific treatment of diseases of the stomach is one of the rarest occurrences in the practice. When the organs of digestion fail to properly perform their task on account of a diseased condition, we usually devote our entire energies to finding some product of the slaughter pen or foreign weed or bark that will do their work for them, instead of making an accurate diagnosis of the diseased condition, and applying scientific treatment that will cure it, thus enabling these organs to do their own work. It takes some trouble and pains to properly examine and treat, by modern methods, diseases of the stomach; but there is nothing in the whole realm of medicine that is more satisfactory in the end.

The particular work which is the occasion of these general remarks is a valuable contribution. There is considerable original work embraced in its pages, and sufficient space has been given to thoroughly present every phase of the subject. A valuable addition to any library. J.

